# Benchmarking Human Protein Complexes to Investigate Drug-Related Systems and Evaluate Predicted Protein Complexes

**DOI:** 10.1371/journal.pone.0053197

**Published:** 2013-02-06

**Authors:** Min Wu, Qi Yu, Xiaoli Li, Jie Zheng, Jing-Fei Huang, Chee-Keong Kwoh

**Affiliations:** 1 School of Computer Engineering, Nanyang Technological University, Singapore, Singapore; 2 State Key Laboratory of Genetic Resources and Evolution, Kunming Institute of Zoology, Chinese Academy of Sciences, China; 3 Data Mining Department, Institute for Infocomm Research, Singapore, Singapore; Koç University, Turkey

## Abstract

Protein complexes are key entities to perform cellular functions. Human diseases are also revealed to associate with some specific human protein complexes. In fact, human protein complexes are widely used for protein function annotation, inference of human protein interactome, disease gene prediction, and so on. Therefore, it is highly desired to build an up-to-date catalogue of human complexes to support the research in these applications. Protein complexes from different databases are as expected to be highly redundant. In this paper, we designed a set of concise operations to compile these redundant human complexes and built a comprehensive catalogue called CHPC2012 (Catalogue of Human Protein Complexes). CHPC2012 achieves a higher coverage for proteins and protein complexes than those individual databases. It is also verified to be a set of complexes with high quality as its co-complex protein associations have a high overlap with protein-protein interactions (PPI) in various existing PPI databases. We demonstrated two distinct applications of CHPC2012, that is, investigating the relationship between protein complexes and drug-related systems and evaluating the quality of predicted protein complexes. In particular, CHPC2012 provides more insights into drug development. For instance, proteins involved in multiple complexes (the overlapping proteins) are potential drug targets; the drug-complex network is utilized to investigate multi-target drugs and drug-drug interactions; and the disease-specific complex-drug networks will provide new clues for drug repositioning. With this up-to-date reference set of human protein complexes, we believe that the CHPC2012 catalogue is able to enhance the studies for protein interactions, protein functions, human diseases, drugs, and related fields of research. CHPC2012 complexes can be downloaded from http://www1.i2r.a-star.edu.sg/xlli/CHPC2012/CHPC2012.htm.

## Introduction

Protein complexes are a form of quaternary structures that are of great importance for understanding cellular organization and functions. They are involved in many essential biological processes, such as the transcription of DNA, the translation of mRNA, signal transduction and other processes. For example, the RNA-Induced Silencing Complex (RISC complex) [1] plays an important role in gene regulation by micro RNAs (miRNA) and in defense against viral infections by incorporating one strand of a small interfering RNA (siRNA) or miRNA. Another example is the RNA polymerase II complex [2], which transcribes genetic information into messages for ribosomes to produce proteins. Moreover, several recent studies have revealed the associations between some specific protein complexes and human disorders. For instance, the Crumbs complex is associated with several human diseases, including blindness and tumor formation [3]. The I
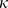
B kinase (IKK complex) is an essential regulator of NF-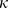
B activation while dys-regulated NF-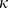
B signaling will lead to various diseases including cancer, chronic inflammation and neurodegenerative diseases [4].

With the recent development of experimental techniques, such as tandem affinity purification with mass spectrometry (TAP-MS), the information and knowledge of these biologically important units become more enriched and are stored in a number of databases. In 2004, a database named PINdb [5], which was compiled from the published literature and existing databases, provides us with many nuclear protein complexes for the first time. Later, MIPS group released a database called CORUM in 2008, which is a collection of experimentally verified mammalian protein complexes [6]. The majority of protein complexes in CORUM originates from human (65%), followed by mouse (14%) and rat (14%). In addition, the HPRD database [7] also provides us with many high-quality human protein complexes that are manually curated. Note that while each database has certain overlaps with others, they can only cover part of the complete set of protein complexes.

As data for protein complexes are identified and classified, various computational applications utilizing those protein complexes have recently been proposed for different purposes. For example, Yang *et al.* [8] integrated data of protein-protein interactions (PPI) and CORUM protein complexes to predict human disease genes. Goh *et al.* [9] also utilized CUROM complexes to calculate Proteomics Signature Profiles (PSP) for cancer patients. PSP profiles can be used for effective clustering of patient samples and is a powerful tool for cancer proteomics. A recent application [10] also utilized CORUM complexes to investigate the relationships between human complexes and drugs. Although CORUM is a credible database, it is still far away from being complete. Hence, it is highly motivated to integrate all the aforementioned databases (i.e., PINdb, CORUM and HPRD) to reflect the current state of knowledge and generate a unique and more comprehensive database for human complexes to enhance the above applications.

Redundancy is a challenging problem which we will definitely encounter when we integrate human protein complexes from different databases. Even records within the same database (e.g., CORUM) are highly redundant [9]. In this paper, we will process those redundant protein complexes to build a non-redundant and comprehensive catalogue called CHPC2012. In particular, we first define the significance score for each protein complex based on their Gene Ontology (GO) term enrichment (using the “biological process” sub-ontology). We then measure the pair-wise similarities between known protein complexes. For two highly similar complexes, we will either discard the one with lower significance score or merge them to form a larger complex, depending on the level of their overlap. To our best knowledge, this is the first attempt to integrate multiple databases and build a unique and comprehensive catalogue for human protein complexes. With the newly generated CHPC2012, we also verified that CHPC2012 is better than individual databases by mapping their co-complex protein associations to existing PPI databases [11].

It is a growing recognition that network-based approaches are suitable to describe the complexity of human diseases and assist the development of new drugs [12]. Recently, Li *et al.* built disease-specific drug-protein connectivity maps using protein interaction networks and literature mining [13]. Nacher and Schwartz constructed networks for protein complexes and drugs. They further investigated the polypharmacological properties by analyzing the topological features of the drug-complex networks [10]. Lee *et al.* established an integrate pharmacological network of proteins, diseases and drugs [14]. In this paper, we introduce an application of our CHPC2012 complexes for drug development by constructing networks at different levels for complexes, drugs and diseases. In particular, drug-drug interactions tend to be co-complex drug pairs (i.e., the drug targets are in the same complex) in the drug-complex network. Disease-specific drug-complex networks, where complexes are enriched with proteins for specific diseases, can provide us valuable information for drug repositioning.

In addition, while experimental methods (e.g., TAP-MS) exist for detecting protein complexes, they have several limitations, e.g., time-consuming, false-negative and false-positive detections. Therefore, computational prediction of protein complexes can fill up the map of protein “complexome” and is thus an interesting topic in bioinformatics. In another application of our CHPC2012 complexes, we evaluate the performance of state-of-the-art computational methods for predicting human protein complexes using CHPC2012 as golden standard. These high quality protein complexes predicted by computational methods may further serve as interesting putative candidates for experimental verifications. Furthermore, analysis on these evaluation results may guide us to streamline future directions in this topic.

## Results

In this section, we will first introduce our CHPC2012 in more details, including the support for CHPC2012 from binary protein interactions. Then, we will show the application of CHPC2012 in drug-related systems. Lastly, we will evaluate the quality of protein complexes predicted by various computational methods using our CHPC2012.

### CHPC2012 for human protein complexes

#### Details for various databases

We applied the Algorithm (see Table 10) to build our CHPC2012 catalogue by integrating the following three databases, namely CORUM, HPRD and PINdb. Table 1 shows some statistics for these three raw databases. We list the number of complexes, proteins and overlapping proteins (i.e., proteins involved in multiple complexes) in the rows 2, 3 and 4 in Table 1. Taking CORUM as an example, it contains 1847 protein complexes (1826 distinct protein complexes), covering 2507 proteins and 1485 out of these 2507 proteins occur in multiple protein complexes. The last row in Table 1 shows the ***redundancy*** of each database, which is measured by the number of redundant pairs. In particular, two complexes with Jaccard similarity higher than the overlapping threshold 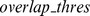 (a pre-defined parameter in the Algorithm) are considered as a redundant pair. For example, the ***redundancy*** for the raw CUROM is 2727, which means there are 2727 redundant pairs among CORUM complexes. Since protein complexes in each raw database themselves are redundant as mentioned previously, we also applied our Algorithm (Table 10) to remove their redundancy.

**Table 1:** Statistics for 3 raw databases.

Raw databases	CORUM	HPRD	PINdb
# complexes	1826	1521	214
# proteins	2507	2738	666
# overlapping proteins	1485	1310	351
Average size	4.79	4.75	8.83
Redundancy	2727	1204	158

In our experiments, two paramters 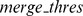 and 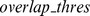 in the Algorithm (Table 10) are set as 0.8 and 0.5 respectively (see more details on the parameter settings in Supporting Information S1). Table 2 shows the statistics of our newly compiled CHPC2012 and the other 3 processed databases. After processed by our Algorithm (Table 10), all the statistics except for the average size of CORUM, HPRD and PINdb are lower. For example, the ***redundancy*** of CHPC2012 is 1, which means that there is only one redundant complex-pair in CHPC2012. At the same time, the other 3 processed databases even have no redundant pairs. In fact, we filtered out 1170 (656 vs. 1826) complexes from CORUM, thereby decreasing the number of covered proteins from 2507 to 1808. We observe that these 1170 filtered complexes (64.07% of 1826 complexes) only cover additional 699 proteins (27.88% of 2507 proteins). These results demonstrate that the Algorithm (Table 10) addressed the redundancy issue very well and the databases processed by this algorithm, unlike those raw databases, no longer suffer from redundant records.

**Table 2:** Statistics for 4 processed databases, including our newly compiled CHPC2012.

Processed databases	CHPC2012	CORUM	HPRD	PINdb
# complexes	1389	656	983	132
# proteins	3065	1808	2386	575
# overlapping proteins	1346	695	908	217
Average size	4.98	5.40	4.54	8.06
Redundancy	1	0	0	0

#### Support from binary interactions

One important application of protein complexes is to derive from them a reference set of co-complex protein-protein associations. Co-complex protein associations here are defined as all the pair-wise links between proteins that belong to the same complex. We can assess the quality of protein complexes by mapping those co-complex protein associations to existing PPI databases. Generally, the set of complexes, which has higher percentage of co-complex associations overlapping with existing PPI databases, has higher quality [11].

To further verify the quality of the complexes in CHPC2012, we generate co-complex protein-protein associations from the above raw and processed databases. Tables 3 and 4 show the generated co-complex protein associations and their overlap with two existing PPI databases, BioGrid and HPRD (note that HPRD provides both PPI data and protein complex data). By comparing these two tables, we have the following three observations. First, for CORUM, HPRD and PINdb databases, the percentage of co-complex associations that overlap with HPRD and BioGrid PPI databases is much higher in the processed databases than in the raw ones. This indicates that the processed complexes have higher quality than the raw complexes. Second, for our CHPC2012, its percentage is slightly lower than those of processed CORUM and HPRD, while much higher than those of raw CORUM and HPRD. Therefore, the quality of our compiled CPHC2012 is guaranteed in coverage of co-complex associations overlapping with the existing PPI databases. Third, the coverage for proteins and complexes is much improved by integrating these databases. For example, Table 2 shows that CHPC2012 contains more complexes and covers more proteins, and moreover Table 4 shows that it has many more co-complex associations that also occur in existing PPI databases. Overall, CHPC2012 is better than other individual databases in balance between quality and coverage based on the above observations.

**Table 3:** Co-complex protein associations for 3 raw databases and their overlap with HPRD and BioGrid protein interactions.

Raw databases	CORUM	HPRD	PINdb
# co-complex pairs	35361	24214	7012
Overlap with HPRD (# pairs)	2475	2843	612
Overlap with HPRD (ratio)	7.00%	11.74%	8.73%
Overlap with BioGrid (# pairs)	4384	4044	1997
Overlap with BioGrid (ratio)	12.40%	16.70%	28.48%

**Table 4:** Co-complex protein associations for 4 processed databases and their overlap with HPRD and BioGrid protein interactions.

Processed databases	CHPC2012	CORUM	HPRD	PINdb
# co-complex pairs	17810	10318	10592	4182
Overlap with HPRD (# pairs)	3245	1925	2486	506
Overlap with HPRD (ratio)	18.22%	18.66%	23.47%	12.10%
Overlap with BioGrid (# pairs)	4853	3061	3356	1470
Overlap with BioGrid (ratio)	27.25%	29.67%	31.68%	35.15%

### CHPC2012 for drug-related systems

#### Druggability of overlapping nodes in protein complexes

Recent studies showed that the overlapping proteins among protein complexes tend to be targets because they are key determinants of the co-operations among complexes [15], [16]. It becomes highly desirable to investigate the druggability of overlapping nodes in four processed databases for complexes. Table 5 shows the number of established drug targets, potential druggable proteins from druggable family [17], druggable proteins in all, the number of overlapping proteins, the ratio of druggable proteins versus the overlapping nodes and the p-value of binomial test for the ratio. Three of the four databases (CHPC2012, CORUM and HPRD) have significantly high ratio of druggable proteins. For example, 26.2% of overlapping nodes in CHPC2012 complexes was druggable proteins, which is significantly higher than expected (2000–3000 druggable proteins in human [17], the expected number of protein-coding genes is about 20500 [18]). Most of the known drug targets are membrane proteins or enzymes while PINdb is a database of nuclear protein complexes. This explains why PINdb has a low ratio of druggable proteins as shown in Table 5.

**Table 5:** Druggable proteins in 4 processed databases.

Processed databases	CHPC2012	CORUM	HPRD	PINdb
# Targets	271	132	204	20
# Potential targets	82	39	66	6
# Druggable proteins	353	171	270	26
# Overlapping nodes	1346	695	908	217
Ratio	0.262	0.246	0.297	0.120
p-value(Binomial test)	2.02E-168	4.04E-71	2.89E-155	9.0E-4

**Figure 1:**
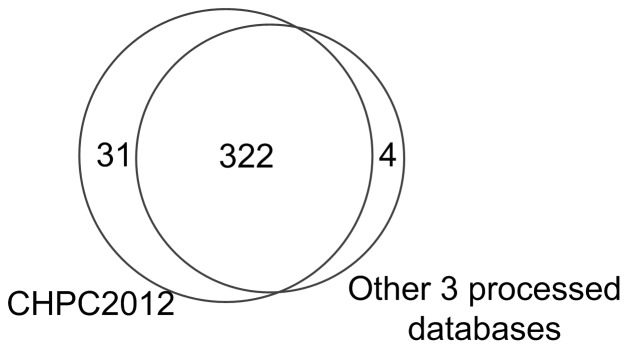
Venn diagram to show the druggable proteins in our CHPC2012 and other 3 processed databases. Figure 1 shows that our CHPC2012 covers almost all the druggable proteins in other 3 databases.

HRPD database has the highest fraction of druggable proteins among overlapping proteins (i.e., 29.7%). However, it is our CHPC2012 that has the most druggable proteins with the lowest p-value. As shown in Figure 1, our CHPC2012 covers almost all the druggable proteins in the other 3 databases. Table 6 shows the common druggable proteins among the 4 databases. For example, CORUM and HPRD have 115 common druggable proteins. As demonstrated above, CHPC2012 is obviously more congruent for investigating druggable proteins than the other 3 databases.

**Table 6:** Overlap of druggable proteins between our CHPC2012 and other 3 processed databases.

Datasets	CHPC2012	CORUM	HPRD	PINdb
CHPC2012	353	170	267	26
CORUM	170	171	115	26
HPRD	267	115	270	24
PINdb	26	26	24	26

#### Network of protein complexes and drugs

We model the relationships between drugs and protein complexes as a network, where both drugs and complexes are nodes, an edge between a drug and a complex indicates that this complex contains at least one target of the drug. We thereafter call this network as drug-complex network. The drug-complex network plays important roles to analyze the features of multi-target drugs and polyphamacological drugs and to unveil novel associations between diseases and protein complexes [10].

The drug-complex network for CHPC2012 complexes consists of 2648 nodes (1835 drugs and 813 complexes) and 9916 edges as shown in Supporting Information S1. The network resembles a scale-free topology. Hubs can be seen among both drugs and complexes as shown in Table 7. We also observe that a small number of drug targets (i.e., proteins in complexes) are used frequently as edges between drugs and complexes. A recent study [10] also built such a drug-complex network based on CORUM complexes, which contains 680 drugs and 739 complexes. Here, by using a more comprehensive database CHPC2012 for complexes, we find many drug hubs that were not observed in [10], such as Guanosine-5′-Diphosphate and Geldanamycin.

**Table 7:** Top drug hubs, complex hubs and targets in complex-drug network.

Drug hubs	Drug degree	Complex ID	Complex degree	Targets	Times used
Adenosine-5′-Diphosphate	166	438	159	CDK2	1620
Vorinostat	65	110	152	HSP90AA1	1260
Flavopiridol	56	460	141	SRC	630
Phosphoaminophosphonic Acid-Adenylate Ester	38	439	141	ESR1	427
Guanosine-5′-Diphosphate	37	462	140	NR3C1	312
Dasatinib	33	528	135	GABRA1	268
DB02754	33	529	135	ADRB2	230
DB07594	33	459	135	F2	200
DB07877	33	357	135	MAPK14	200
Geldanamycin	33	461	135	CCNA2	198

However, some drugs and targets might be over-counted. For example, suppose that three complexes (A, B and C) share a protein D which is a target of drug E, so drug E is counted 3 times (A–E, B–E, C–E) and target D is also used 3 times. To avoid the over-counting problem, we should delete the repeats, i.e., both drug E and target D should be counted only once. Table 8 shows that the resulting hubs are thus changed — 5 new drug hubs are listed, namely NADH, Alpha-D-Mannose, Adenosine triphosphate, L-Glutamic Acid and Myristic Acid. Taking NADH as an example, it is the second top drug hub and its degree is 29. It is an FDA-approved nutraceutical drug and is useful in treating Parkinson's disease, chronic fatigue syndrome, Alzheimer's disease and cardiovascular disease. Hence, it is not surprising that NADH is a hub because it is a broad-spectrum nutraceutical drug. The targets are also quite different between Tables 7 and 8. After removing repeats, the most frequently used targets in Table 8 include GABAA receptros (GABRA1, GABRA2, GABRB2, GABRG2), which have benzodiazepine sites and anxiolytic effect, CDK2, which is a kind of protein kinase and anti-cancer target, and F2 (Coagulation factor II), which is a target for anticoagulant drugs.

**Table 8:** Top drug hubs and targets after removing repeats.

Drug hubs	Drug degree	Targets	Times used
Adenosine-5′-Diphosphate	33	GABRA1	185
NADH	21	CDK2	168
Guanosine-5′-Diphosphate	16	F2	100
Alpha-D-Mannose	16	GABRA2	93
Phosphoaminophosphonic Acid-Adenylate Ester	13	GABRB2	72
Dasatinib	12	GABRG2	68
Adenosine triphosphate	12	CCNA2	66
L-Glutamic Acid	12	DRD2	64
Flavopiridol	11	ESR1	61
Myristic Acid	9	HSP90AA1	55

As previously reported in Table 5, CHPC2012 has a higher coverage of druggable proteins. The drug-complex network for CHPC2012 also cover more complexes and drugs than those for a single database such as CORUM. In addition, we provide a more accurate estimation of network characteristics (e.g., degree). Hence, we believe that the drug-complex network for CHPC2012 would be helpful for us to uncover more potential associations between protein complexes and drug-related systems.

#### Drug-complex network and drug-drug interactions

**Figure 2:**
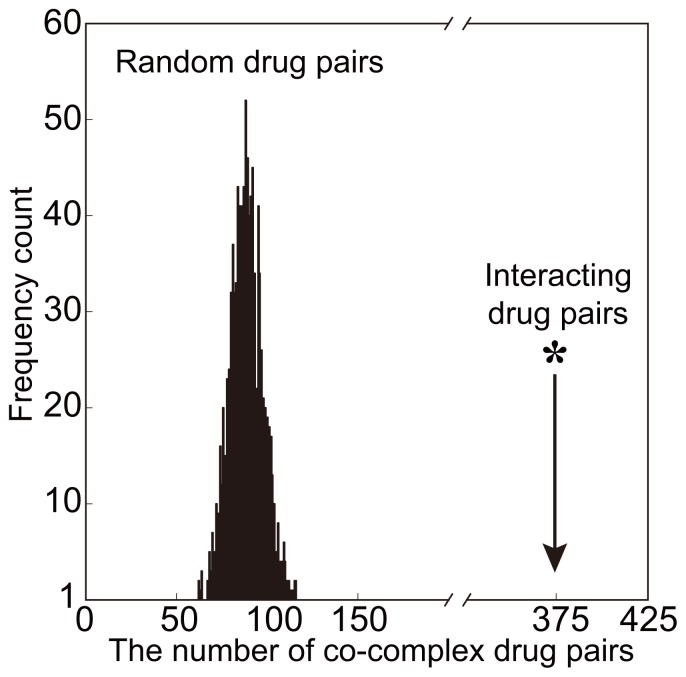
The interacting drug pairs are likely to be co-complex drug pairs. Figure 2 shows that there are 375 co-complex drug pairs among 1901 known drug-drug interactions. Figure 2 also shows the distribution of the numbers of co-complex drug pairs in 1000 random tests.

Drug-drug interaction is a situation where a drug affects the activity of another drug when taken together. In our drug-complex network, we found several drugs are interacting with the same complex (we call these drugs as co-complex drug pairs). Our hypothesis is that drug-drug interactions tend to be co-complex drug pairs. To verify this hypothesis, we perform the following random test. Among 1901 known drug-drug interactions, there are 375 co-complex drug pairs. We also generate 1901 random drug pairs for 1000 times. In this case, the average number of co-complex drug pairs in these random pairs is only 88. Figure 2 also shows the distribution of the numbers of co-complex drug pairs in these 1000 random tests. These results validate the above hypothesis effectively. Therefore, the drug-complex network provides a new approach to *in silico* prediction of drug-drug interactions.

#### Disease-specific complex-drug networks and drug repositioning

We link phenotypes or diseases to protein complexes based on their enrichment in these complexes (see the Equation 3 in the Methods section). Disease-specific complex-drug networks consist of a specific disease, all the complexes connecting to this disease and all the drugs connecting to these disease-related complexes. Therefore, we assume that all the drugs in a disease-specific complex-drug network are associated with the given disease. Here, drug-disease associations mean that these existing drugs may be potential treatments for the diseases and the disease-specific complex-drug networks can thus be utilized for drug repositioning [19]. Using our CHPC2012 complexes, we managed to predict 1400 novel drug-disease associations, involving 600 drugs and 62 diseases.

We applied the literature mining techniques to verify the above identified drug-disease associations [20], [21]. In particular, a drug-disease association is considered to be verified if its drug and disease share at least one PubMed record (see the Methods section for more details). Verified drug-disease associations are supposed to have higher reliability than those unverified association. Meanwhile, unverified associations also provide novel knowledge for further investigation. In our experiments, there are 1103 out of 1400 drug-disease associations whose drugs and diseases have PubMed records. 371 out of 1103 associations, covering 223 drugs and 43 diseases, are verified associations while the remaining 732, covering 420 drugs and 54 diseases, are novel drug-disease associations (all 1103 predicted drug-disease associations can be found on our website).

**Figure 3:**
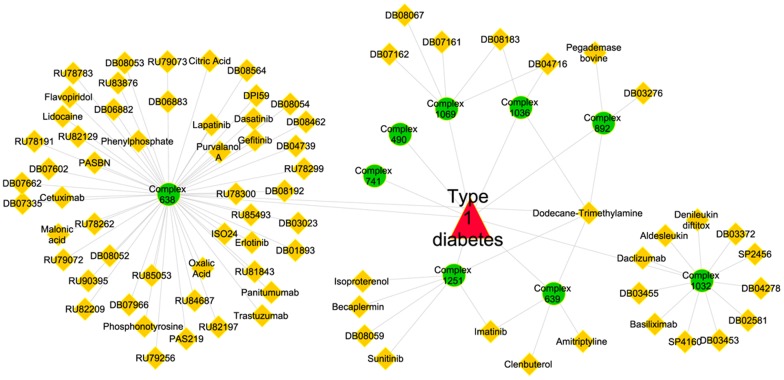
The drug-complexes network in Type 1 diabetes. Figure 3 shows the drug-complexes network in Type 1 diabetes. Complexes in figure 3 are represented by circles, drugs by diamonds and disease by triangle.

The association between the drug Imatinib and type 1 diabetes is a verified example predicted in our disease-specific complex-drug networks. Type 1 diabetes is an autoimmune disease that destructs insulin-producing beta cells of the pancreas with subsequent lack of insulin and leads to increased blood and urine glucose. The complexes linked to Type 1 diabetes as shown in Figure 3 can be divided to three categories: (1) a group of complexes (complex 638, 639, 892, 1036, 1069, 1251) interconnected by shared drugs, (2) a single complex (complex 1032) associated to 11 drugs and (3) complexes involved in the disease but not linked to any drugs (complex 490, 741). This highlights a certain bias of current pharmacological approaches, which tend to focus on a few targets for which multiple drugs have been developed. Meanwhile, Imatinib connects with complex 639 and complex 1251 in our complex-drug network and is a tyrosine kinase inhibitor for treating cancer. In our analysis, this drug is predicted to be promising for treating Type 1 diabetes. In fact, our discovery agrees with experimental research that Imatinib is a new class of drug to cure type 1 diabetes by Louvet *et al.* [22], providing new insights into possible solutions for such complex diseases.

### CHPC2012 for evaluating predicted protein complexes

Currently, there are various computational approaches for predicting protein complexes from protein interaction data, e.g., MCODE [23], MCL [24], DPClus [25], CFinder [26], DECAFF [27], IPCA [28], COACH [29], CMC [30], C2S [31] and so on. A comprehensive survey of such methods can be found in the review paper [32].

In this work, CHPC2012 will be utilized to evaluate the quality of protein complexes predicted by five of the above approaches (e.g., MCODE [23], MCL [24], DPClus [25], CFinder [26] and COACH [29]). We exploited two PPI datasets, namely HPRD [7] and BioGrid [33]. HPRD data consist of 9454 proteins and 36868 protein-protein interactions and BioGrid data consist of 11120 proteins and 55014 protein-protein interactions. We used various measures to evaluate the performance of the above approaches, e.g., precision, recall, F-measure [29], [34], sensitivity, PPV and accuracy [31], [35].

Table 9 shows the detailed comparative results of the various computational detection methods on the HPRD data and the BioGrid data, respectively. For each detection method, we have listed the number of predicted complexes (# complexes), the number of proteins covered by the predicted complexes (# covered proteins), the number of predicted complexes which match at least one real complex (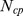) and the number of real complexes that match at least one predicted complex 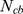.

**Table 9:** Results of various approaches using HPRD and BioGrid PPI data.

Database	Algorithms	COACH	MCODE	MCL	CFinder	DPClus
HPRD	# Complexes	1751	152	3129	418	640
	# Covered proteins	3765	1088	9454	4288	2543
	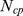	676	61	534	121	285
	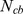	689	116	694	149	486
BioGrid	# Complexes	2055	151	2541	219	442
	# Covered proteins	4851	1347	11120	5547	2357
	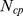	660	50	355	43	192
	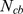	655	77	479	50	363

For example as shown in Table 9, MCODE predicted 152 complexes, of which 61 match 116 real complexes. These 152 predicted complexes cover 1088 out of 9454 proteins in HPRD database. It is observed that the complexes predicted by MCL cover all the proteins in the PPI networks while those by all the other methods only cover a fraction of proteins. This is because MCL assigned every protein in the PPI network into its predicted complexes while all the other methods only assigned those highly interactive proteins into the predicted complexes. In addition, we notice that MCODE and CFinder predict just a small number of protein complexes while COACH and MCL predict quite a large number of protein complexes (the number of protein complexes predicted by DPClus is moderate).

**Figure 4:**
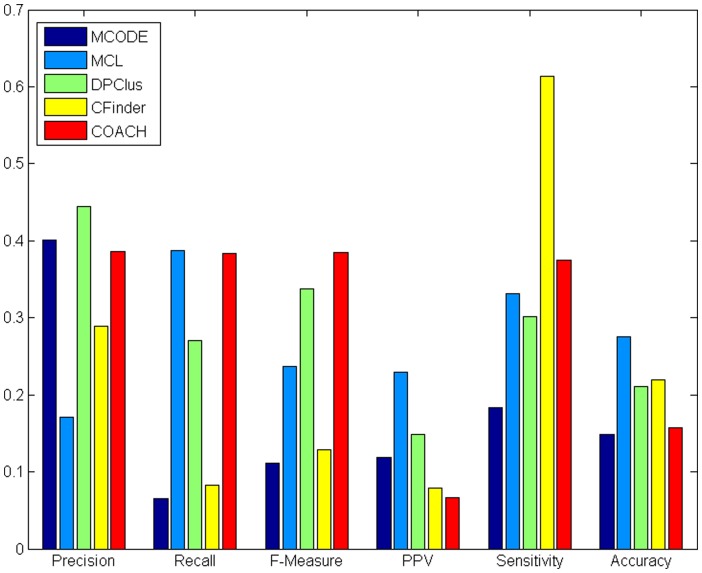
Comparative performance of existing methods in terms of various evaluation metrics for HPRD data. Figure 4 shows comparative performance of existing methods in terms of various evaluation metrics for HPRD data.

**Figure 5:**
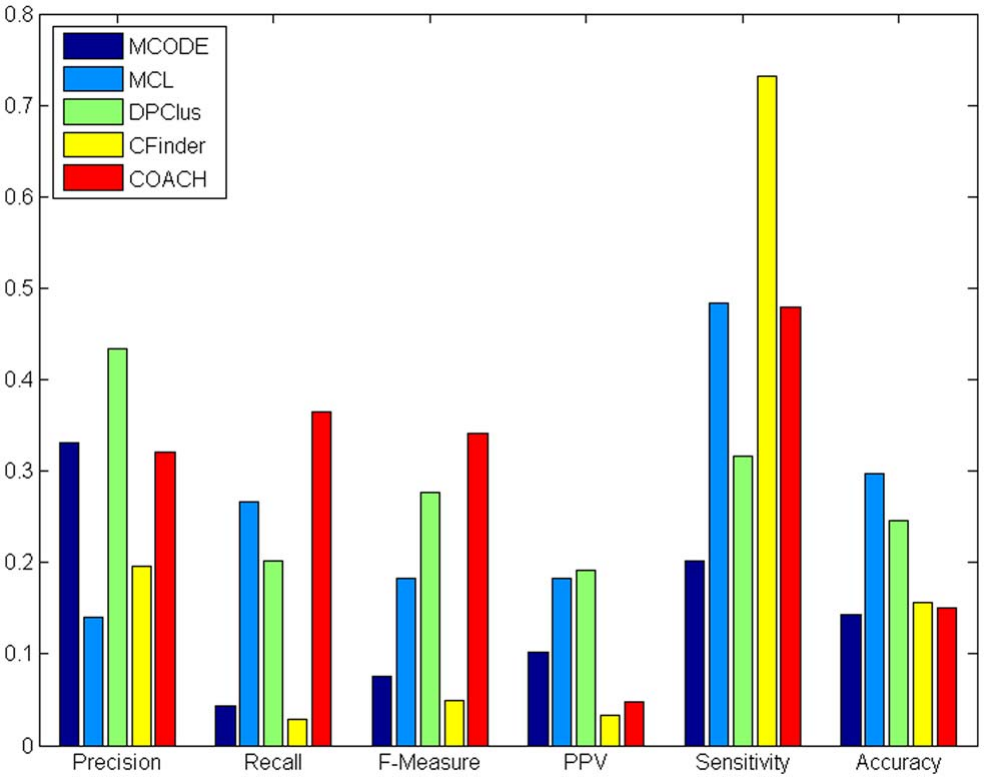
Comparative performance of existing methods in terms of various evaluation metrics for BioGrid data. Figure 5 shows comparative performance of existing methods in terms of various evaluation metrics for BioGrid data.

Figures 4 and 5 show the overall comparison results of existing methods in terms of various evaluation metrics for HPRD and BioGrid data, respectively. In both Figures 4 and 5, we observe that MCODE was able to achieve the second highest precision (lower than DPClus), indicating that protein complexes predicted by MCODE have high quality. However, as pointed in Table 9 above, both the number of predicted complexes (only 152 for MCODE) and real complexes that can be matched by predicted complexes (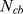 is only 116 for MCODE) are very small. Therefore, MCODE has a low recall and F-measure values.

We also noticed that CFinder attained an unusally higher sensitivity than other methods. This is actually because it predicted a huge cluster with too many proteins (e.g., the biggest cluster predicted from HPRD contains 3434 proteins). In this case, all the proteins in those real complexes were pretty much covered by this very big cluster. Meanwhile, COACH achieved the highest F-measure due to its balanced precision and recall in both Figures 4 and 5. Overall, the performance of these approaches in terms of F-measure is in the following order: COACH and DPClus have relatively high F-measure, MCL is moderate while MCODE and CFinder have low F-measure.

After showing the comparative results of various methods in the above table and figures, we can find that these results are consistent with those collected for the model organism yeast *Saccharomyces cerevisiae* [32]. For example, MCODE has high precision and low recall when mapping its predicted complexes to both real yeast and human complexes. In addition, various methods are rated similarly based on their performance for yeast and human. As we know, the yeast complexes [11] used for evaluation are well curated and categorized. Based on the consistency of evaluation results between yeast and human, it is once again confirmed to some extent that our CHPC2012 catalogue provides comprehensive human protein complexes with high accuracy and coverage for future research and applications.

However, a difference between the results of human and yeast is that the performance of each individual method is much lower in human than in yeast. For example, many methods including MCL, DPClus and COACH achieve a recall higher than or close to 0.6 on Krogan PPI data while all of them have a recall lower than 0.4 on both HPRD and BioGrid datasets. The recall of MCL on BioGrid data is even lower than 0.3. In addition, all the methods used in the paper have very low PPV values, which motivated the following strategies to improve the performance of protein complex prediction for human. First, we may improve the quality of our inputs, i.e., the PPI data. Due to the high false positive and false negative rates, it would be necessary to assess the reliability of PPI data used for protein complex detection [36]. Second, we may further integrate the topological properties of human proteins in various biological networks (e.g., PPI networks) as well as their genomic characteristics (e.g., gene expression profiles) and then devise novel algorithms to cover those real complexes that can't be matched by current methods. Last and the most importantly, we admit that our CHPC2012 catalogue is still far from complete. We should keep on improving its quality with more biological insights into these protein complexes to gradually complete the map of protein “complexome”. For example, in order to better integrate those complexes from various data sources, we should take the reliability of these data sources or their detection methods into considerations.

### Discussion and Conclusions

Protein complexes are key entities to perform cellular functions and associate with specific human diseases. However, they are highly redundant within and across various existing databases, such as CORUM and HPRD. In this paper, we processed these redundant complexes and compiled a non-redundant catalogue for human protein complexes called CHPC2012. CHPC2012 is verified to be a high quality set of protein complexes as it has a high coverage for proteins and protein complexes, as well as protein-protein interactions in existing PPI databases. It reveals extensive advantage for applications in drug development, especially in the drug-target prediction, drug-drug interactions, and drug repositioning. CHPC2012 also provides a multidimensional view of associations among biological components, including drug-complex networks and disease-specific complex-drug networks. In addition, CHPC2012 can be used as a golden standard benchmark to evaluate the performance of various methods that are designed to predict protein complexes from human PPI data. The evaluation results also provide several useful hints to fill up the current map of human protein “complexome”.

Translational bioinformatics is dedicated to translate scientific discoveries into better therapeutic outcomes through integrative methods [37]–[39]. Our analysis based on the CHPC2012 complexes is an attempt for translational bioinformatics to improve our understanding of complex diseases and their therapies. For example, overlapping proteins among multiple complexes tend to be drug targets. The construction of drug-complex networks makes it much easier to speculate drug hubs (i.e., multi-target drugs) and analyze drug-drug interactions. Drug-drug interactions can lead to severe side effects and in some cases have resulted in early termination of development [40]. The prediction of drug-drug interactions in *silico* will provide guideline for in *vitro* and in *vivo* studies. Furthermore, disease-specific drug-complex networks (taking disease-gene associations into consideration) reveal novel associations between drugs and diseases (i.e., drug repositioning). With these achievements of CHPC2012 for drug development, we reasonably expect that broader applications of CHPC2012 will be conducted for translational bioinformatics in the future.

We applied literature mining techniques to verify our drug repositioning results. Around a third of our predicted drug-disease associations have at least one literature support, which suggests our prediction is reliable and believable. Meanwhile, two thirds of the associations are completely new discoveries, showing the novelty of our analysis. However, we must be very cautious with the computational verification of drug repositioning. A co-occurrence of a drug and a disease sometimes implies that the drug might cause the disease instead of cure it (i.e., a false positive prediction). Therefore, more sophisticated literature mining techniques (e.g., searching more keywords like “treatment”, “cure” and so on) are needed for a more precise computational verification. Although such false positive predictions cannot be ignored, our analysis nonetheless can provide valuable information for drug repositioning.

Lastly, Gene Ontology also provides some information for protein complexes in the “cellular component” sub-ontology. We didn't utilize the complexes in GO to compile our CHPC2012 for the following reasons. First, we define the significance scores for known complexes based on their GO term enrichment, which would bring bias for complexes in GO. Second, some complexes in GO are computationally inferred and they may not be accurate. Third, the support from binary interactions shows that complexes in GO have much lower quality than those in other databases as shown in Supporting Information S1 — GO complexes have the lowest percentage of co-complex protein associations that also occur in existing PPI databases. However, the hierarchical organization of GO complexes inspires us to construct a more accurate ontology for protein complexes. Some complexes, which are considered to be redundant in this work, may be distinct in reality and perform functions at different levels. A more accurate ontology for complexes would address the redundancy issue better than our current CHPC2012. Another future study is to predict disease-related genes and protein complexes by integrating our CHPC2012 complexes and some other data sources, such as gene expression data from next-generation sequencing of cancer cells.

## Methods

### Compilation of the CHPC2012 catalogue

After human protein complexes from individual databases are collected, we compile them to construct a more comprehensive catalogue for further reference. However, many of those collected complexes may overlap with one another. In order to process these redundant complexes, we define a significance score for each complex. Since proteins within the same complex tend to cooperate with each other to perform a common function, the significance score of a complex here will show the extent of its functional enrichment. In particular, a protein pair can have a semantic similarity based on their GO annotations. The significance score of a complex 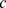, 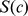 in equation 1, is the average semantic similarity for all protein pairs within this complex. Specifically, the semantic similarity between proteins 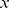 and 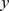, 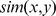 in equation 1, is calculated using the method proposed in [41]. In addition, the overlap/similarity between two complexes is measured by the Jaccard coefficient in equation 2.
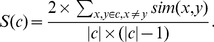
(1)

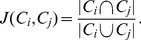
(2)We have to be cautious to process the redundant complexes. For two complexes 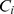 and 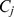, we will perform the following operations based on their Jaccard similarity. If they are highly similar (i.e., 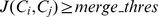, where 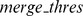 is a pre-defined parameter), we will merge them because they are almost identical. If they are relatively similar (i.e., 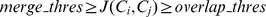, where 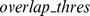 is another parameter), we will not merge them because the merged complex would be arbitrary and may not reflect a true biological unit. In this case, keeping the one with larger significance score is a safe decision. If two complexes are not similar (i.e., 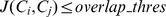), we will of course keep both of them.

The above strategies are presented in the Algorithm (Table 10), which is similar to the CMC algorithm [30] (clustering based on maximal cliques). CMC algorithm was originally designed to merge and remove highly overlapped cliques to predict protein complexes from human PPI data. In Line 4, two complexes 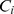 and 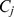 are considered to be redundant if their Jaccard coefficient is larger than 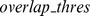. In Lines 5–6, 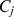 will be merged in 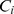 if their Jaccard coefficient is larger than 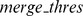 and 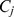 will be discarded otherwise. Finally, the output of the Algorithm is our new catalogue CHPC2012.

**Table 10:** The Algorithm to Construct the *CHPC*2012 Catalogue.

**Input:** *C*, the set of raw human protein complexes;
*Overlap_-_thres*, the overlapping threshold;
*Merge_-_thres*, the threshold for merging;
**Output:** *CHPC*2012 catalogue of human protein complexes;
1: Calculate the significance score of each complex and rank all the complexes in the descending order of significance scores, denoted as 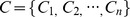 //the significance score for a complex is defined in equation (1).
2: **for all** *C_i_* 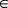 * C*
3: **for all** *C_j_* 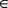 * C* and *j>i*
4: **if** *J*(*C_i_;C_j_*)*>overlap_-_thres* **then**
5: **if** *J*(*C_i_;C_j_*)*>merge_-_thres* **then**
6: 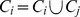
7: *C* = *C−C_j_*
8: **end for**
9: **end for**
10: return *C* as our *CHPC*2012 catalogue.

### Disease-gene associations and disease-complex associations

Disease-gene associations were collected from 118 GWAS articles [42] as well as the GWAS catalog [43] (March, 2012). Fisher's meta p-value was calculated to combine p-values when the same disease-gene association was reported in different studies. We only keep associations with p-value lower than 10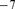.

Based on the above collected disease-gene associations, we extract disease-complex associations using the following hypergeometric distribution [32] in Equation 3.
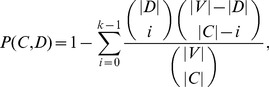
(3)where a complex 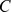 contains 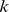 proteins associating with the disease 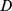. 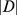 is the number of proteins associating with the disease 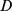 while 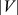 is the number of human proteins in our CHPC2012 catalogue. All the disease-complex associations with 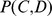 less than 0.05 are considered to be significant [44] and thus kept for us to construct the disease-related complex-drug network.

In addition, the drug targets and drug-drug interactions were downloaded from Drugbank (Release 3.0) [45] in March, 2012.

### Computational verification for drug repositioning

By constructing disease-specific drug-complex networks, we are able to predict novel drug-disease associations for existing drugs (i.e., drug repositioning). Here, we briefly introduce the computational verification for those predicted drug-disease associations.

As we know, MeSH (Medical Subject Headings thesaurus) is a comprehensive controlled vocabulary for the purpose of indexing scientific articles and it can also serve as a thesaurus that facilitates searching in PubMed. To search drugs and diseases in PubMed, we will map them to MeSH terms. For example, 315 drugs and 59 diseases have MeSH terms (there are 600 drugs and 62 diseases in 1400 predicted drug-disease associations). They can be automatically linked to PubMed records (i.e., PubMed id). For those drugs or diseases without MeSH terms, we will directly use their names for searching in PubMed. As such, 550 drugs and 59 diseases in 1103 drug-disease associations have at least one PubMed record. Finally, if a drug and a disease have a co-occurrence in the PubMed record (i.e., they share a common PubMed id), they are considered to be a verified drug-disease association. Otherwise, they are considered as a novel drug-disease association.

## Supporting Information

Supporting Information S1 **Supporting Information S1. Includes supplementary figures and tables.**
(PDF)
