# Supplementary material for: Benchmarking Human Protein Complexes to Investigate Drug-Related Systems and Evaluate Predicted Protein Complexes
Source: PLoS One. 2013 Feb 6;8(2):e53197. doi: 10.1371/journal.pone.0053197 (PMC3566178; doi:10.1371/journal.pone.0053197)
Supplement: Supporting Information S1 — Includes supplementary figures and tables. (PDF) [file pone.0053197.s001.pdf]

Benchmarking Human Protein Complexes to Investigate Drug-Related Systems and Evaluate Predicted Protein Complexes

Min Wu, Qi Yu, Xiaoli Li, Jie Zheng, Jing-Fei Huang and Chee-Keong Kwoh

1 A drug-complex network

The drug-complex network for our CHPC2012 complexes consists of 2648 nodes, including 1835 drugs and 813 complexes, and 9916 edges as shown in the following Figure S1. In this figure, orange diamonds represent drugs and green circles are protein complexes in CHPC2012.

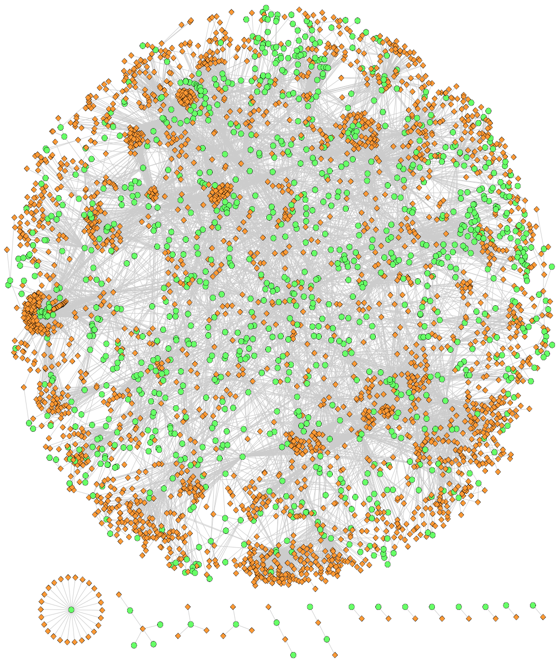

Fig. S1. The drug-complex network for complexes in CHPC2012.

2 Human protein complexes in Gene Ontology (GO)

We processed the "cellular-component" sub-ontology of GO and collected 486 complexes for human. Table S1 shows the co-complex associations for these 486 complexes in GO, as well as three aforementioned databases, i.e., CORUM, HPRD and PINdb. We can find that the complexes in GO have the lowest fraction of co-complex protein associations in HPRD and BioGrid PPI databases. This indicates that the raw set of GO complexes has the lowest quality.

Table S1. Co-complex protein associations for 4 raw databases and their overlap with HPRD and BioGrid protein interactions.

Raw databases	CORUM	HPRD	PINdb	GO
# co-complex pairs	35361	24214	6943	22990
Overlap with HPRD (# pairs)	2475	2843	614	1382
Overlap with HPRD (ratio)	7.00%	11.74%	8.80%	6.01%
Overlap with BioGrid (# pairs)	4384	4044	1992	2000
Overlap with BioGrid (ratio)	12.40%	16.70%	28.69%	8.70%

We also processed the GO complexes with our Algorithm 1 (see the main manuscript). Table S2 shows that the quality of GO complexes is not improved in terms of the percentage of co-complex associations in existing PPI databases. However, the quality of complexes in other 3 databases (CORUM, HPRD and PINdb) is improved significantly as shown in Table S2. Note that CHPC2012 is obtained by integrating CORUM, HPRD and PINdb databases. The results in Tables S1 and S2 demonstrate that the quality of GO complexes is not good and this is the main reason we did not include GO complexes to build our CHPC2012.

Table S2. Co-complex protein associations for 5 processed databases and their overlap with HPRD and BioGrid protein interactions.

Processed databases	CHPC2012	CORUM	HPRD	PINdb	GO
# co-complex pairs	17810	10318	10592	4182	22819
Overlap with HPRD (# pairs)	3245	1925	2486	506	1375
Overlap with HPRD (ratio)	18.22%	18.66%	23.47%	12.10%	6.02%
Overlap with BioGrid (# pairs)	4853	3061	3356	1470	1994
Overlap with BioGrid (ratio)	27.25%	29.67%	31.68%	35.15%	8.74%

3 Parameter Settings

In the Algorithm 1 in our main manuscript, there are two parameters namely *overlap_thres* and *merge_thres*.

For the parameter *overlap_thres*, it is used to determine whether two complexes are redundant (i.e., they can match each other). In a previous study [1], this parameter is set as 0.5. Therefore, we have also followed their suggestion and set it as 0.5 in our experiments. Let us give a specific example for this parameter. Suppose that there are two complexes and both of them have 8 proteins, they can be considered to be matching when the number of proteins in common between them is at least 6 (if the intersection has 6 proteins, the union will have 10 proteins and the Jaccard coefficient is 0.6; if the intersection has 5 proteins, the union will thus have 11 proteins and the Jaccard coefficient is 0.455) [1].

For the parameter *merge_thres*, it is used to determine whether to merge two redundant complexes or not. We are cautious to process those redundant complexes. If the value of the parameter *merge_thres* is set too low, then we may arbitrarily merge two different complexes as long as they share some protein components, which could generate false positive protein complexes.

As introduced in the main manuscript, co-complex protein associations are defined as all the pair-wise links between proteins within the same complexes. We can further assess the quality of a set of protein complexes by mapping its co-complex associations to existing PPI databases — A set of protein complexes that have higher percentage of co-complex associations overlapping with existing PPI databases tend to have higher quality [2].

As we know, CHPC2012 will have a different number of complexes when we use different values for *merge_thres*. Following your comments, we have performed additional experiments to investigate how the values of *merge_thres* affect the quality of final protein complex list. In particular, Figure S2 shows the percentage (ratio) of co-complex associations of CHPC2012 that overlaps with existing PPI databases, i.e., HPRD and BioGrid. The overall trend for the ratio curves is quite obvious—the ratio (i.e., the quality of CHPC2012) increases as the values of *merge_thres* incresase. However, we can still have the following two additional observations from Figure S2.

Firstly, the ratio increases quite rapidly as we increase the value for *merge_thres* when *merge_thres* is small (i.e., in [0.5, 0.65]). Smaller *merge_thres* leads to even lower quality of CHPC2012, indicating that the merging of complexes is indeed arbitrary using small values for *merge_thres*.

Secondly, the ratio increases much slowly when *merge_thres* is relatively large (i.e., in [0.7, 0.95]). This indicates that the quality of CHPC2012 is stable and guaranteed after *merge_thres* becomes big. Therefore, we prefer to set *merge_thres* in the range [0.7, 0.95]. On the other hand, the number of co-complex associations and proteins covered by CHPC2012 decreases as we increase the value for *merge_thres*. This demonstrates that the coverage of CHPC2012 will decrease as *merge_thres* increases. To balance the coverage and the ratio of co-complex associations, we finally set *merge_thres* as 0.8 in our experiments.

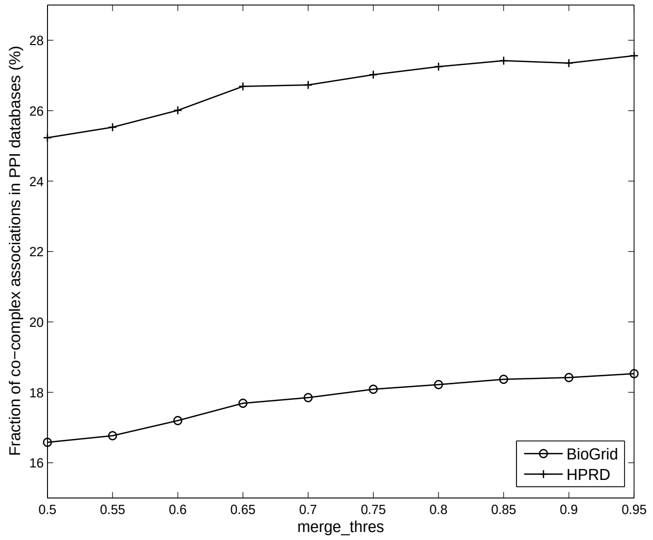

Fig. S2. The fraction of co-complexes associations in CHPC2012 overlapping in existing PPI databases, using different values for *merge_thres*.

Table S3. Details of CHPC2012 collected by using different values for the parameter *merge_thres*.

merge_thres	0.5	0.55	0.6	0.65	0.7	0.75	0.8	0.85	0.9	0.95
#complexes	1507	1411	1406	1412	1407	1388	1389	1390	1390	1389
#proteins	3298	3152	3140	3105	3093	3066	3065	3040	3037	3033
#overlapping proteins	1556	1442	1428	1393	1378	1350	1346	1335	1336	1326
#co-complex pairs	22902	20829	20128	18978	18695	17987	17810	17405	17328	17066
Overlap with HPRD (#pairs)	3796	3493	3463	3357	3337	3253	3245	3198	3191	3162
Overlap with HPRD (ratio)	16.58%	16.77%	17.20%	17.69%	17.85%	18.09%	18.22%	18.37%	18.42%	18.53%
Overlap with BioGrid(#pairs)	5779	5317	5235	5066	4997	4860	4853	4772	4740	4703
Overlap with BioGrid(ratio)	25.23%	25.53%	26.01%	26.69%	26.73%	27.02%	27.25%	27.42%	27.35%	27.56%

References

1. G. M. Liu, H. N. Chua, and L. Wong. Complex discovery from weighted ppi networks. *Bioinformatics*, 25(15):1891–1897, 2009.
2. S. Pu, J. Wong, B. Turner, E. Cho, and S. J. Wodar. Up-to-date catalogues of yeast protein complexes. *Nucleic Acids Research*, 37(3):825–831, 2009.
